# On Artificial Anus

**Published:** 1822-02

**Authors:** Daniel Pring

**Affiliations:** Surgeon.


### CHIRURGERY.

### ART. V.—*On Artificial Anus.* By DANIEL PRING, Surgeon.

THE object of my present communication is to give the sequel of a case of artificial anus, an account of which was published in the London Medical and Physical Journal for January 1821. As the case alluded to may probably have escaped altogether the recollection of your readers, it may not be amiss to recapitulate some particulars of its history. The symptoms, from the beginning, consisted of acute pains in various parts of the abdomen, with occasional discharges of blood from the rectum. The patient, who was between sixty and seventy years of age, suffered, sometimes, constipations, which were with great difficulty overcome. She was constantly under the necessity of taking aperient medicines, in order to procure fluid stools, as no others could pass. At length it became impossible to procure evacuations of any sort: the rectum was supposed to have become completely impervious; and, on the twelfth day from the commencement of the last obstruction, an opening was made in the colon, near its sigmoid flexure, with a view to the formation of an artificial anus. The report, formerly published, goes on to state, that the wound sloughed to a great extent, that eventually it healed, and the lady left Bath in tolerably good health. She returned to her residence in Gloucestershire, and for a short time suffered no very great inconvenience from her disease: the feces were voided with perfect ease from the artificial anus; she walked out, and went to church. By degrees, however, the acute pains, which she had formerly felt in the abdomen, returned: small quantities of blood, mixed with fetid matter, were sometimes discharged from the lower part of the opening in the colon; and similar discharges occurred, also, from the natural anus. These symptoms progressively increased, and her general health became proportionally impaired. At length she was confined entirely to her bed. The feces continued to be evacuated, without difficulty, from the side: the discharges of blood and pus became more copious; she was distressed by frequent vomiting; her appetite failed her, she became emaciated, and, worn out by long-continued disease, died on the 25th of October; having survived the operation a year and nearly four months.

On the 28th of October, assisted by Mr. George Skinner, I made an examination of the body. It was observed, that the peritoneum, omentum, and viscera, generally, were of a darker colour than is natural, as if these parts had suffered, in some degree, from inflammation: there were, however, no preternatural adhesions, nor did it appear that the inflammation had produced effusion of lymph from the external surfaces. The colon, in the situation of the wound, was firmly united to the parietes of the abdomen; and the opening, which had never shown a disposition to contract, was sufficiently capacious to admit a substance twice the size of the larger rectum-bougie. This large opening was not very evident during the life of the patient: it always appeared closed, and this portion of the bowel much corrugated. The descending colon was continued about four inches below the wound, and its appearance was not remarkably unhealthy. The principal disease commenced at the top of the rectum: this gut was, from its connexion with the colon, almost of a cartilaginous firmness; it was very much thickened, and its calibre, of course, greatly diminished. Posteriorly, the rectum was firmly adherent to the sacrum: at about its middle portion, in front, it was destroyed, for the space of perhaps two inches in length, by ulceration; below this part, it preserved the cartilaginous structure, and was so much thickened, that its canal barely admitted the introduction of an urethra-bougie. The rectum was not continued on to the natural anus, but appeared totally destroyed, by sloughing and ulceration, to the extent of two inches before it reached this part. The uterus and bladder appeared not to have suffered, in any degree, from disease. The liver, spleen, &c. were also healthy. The external coat of the stomach, as well as that of the small intestines, appeared darker than usual: their internal surfaces were also examined, but no unequivocal traces of disease were observed. As there could be little doubt but that some portion of the rectum was completely closed at the time of the operation, it is probable that this portion was that nearest the colon, which was subsequently found destroyed by ulceration, since the gut, in other parts, was sufficiently pervious to have admitted the escape of fluids, of which there was no discharge from the natural anus for some months after the operation.

It will scarcely be doubted that the obstruction, in this case, was occasioned by a scirrhous state of the rectum, which commenced near its connexion with the colon, and gradually occupied its whole extent. This state of disease went into the ulcerated stage, and the patient died from the progress of cancer, by the same influence upon the constitution, and perhaps in about the same time, as if a similar disease had occupied any other part.

With respect to the eligibility of resorting to this operation in future instances, of which this case has, in some degree, qualified me to judge, this question is to be considered on two grounds: the first, is in reference to the probability of success; the second, regards the inconveniences of an anus in such a situation. There is no doubt but the operation may, under many circumstances, be a probable means of prolonging life. The principal danger to be apprehended from it, is that of inflammation of the peritoneum and intestines: this, indeed, may appear a formidable risk; but it will be so to those only who are but indifferently acquainted with the powers of purgatives, and with a mode of employing them which is deduced from an extensive experience, and an accurate observation of their effects. Peritonitis may generally be prevented, when there is reason to apprehend it, by the free use of calomel, jalap, salts, senna, perhaps emetic tartar and ipecacuanha: but these means, although they will go a great way towards it, may be unequal to arrest its progress, if this condition of disease be already established.

The formation of an artificial anus, as is proved by the cases of Duret and of Mr. Freer,* is applicable to children born with an imperforate anus, in whom the rectum cannot be found by an opening in the usual situation. It is also applicable to a scirrhous state of the rectum; but, in this case, it will be regarded only as a palliative, or as a means of prolonging life for a short period, by obviating one effect of an incurable disease. In obstructions of the rectum or colon, which are merely mechanical, and where no disease exists which, in its nature, is inevitably fatal, the operation is a resource of a less exceptionable character. Cases of this last description, although of rare occurrence, are occasionally met with: it is, however, probable, from the frequent obscurity of visceral disease, that the precise nature or seat of the obstruction may not always be accurately discovered; which, in contemplating a project of this sort, appears to be a matter of some importance. But, although it may not, in all cases, be possible to discriminate the precise state of

* See *London Med. and Phys. Journal*, for January 1821.

the obstruction, it is sufficient, in order that the case may admit the relief of this operation, if it can be ascertained that it exists in the large intestines. If the obstruction is in the rectum, it may be discovered by the finger or a bougie ; if in the colon, the distention of this gut may most probably be felt by a careful examination of the abdomen; and *an opening in any portion which is felt to be distended*, will certainly be above the seat of the obstruction ; or, more properly, on that side of the obstructed portion of the colon which is nearest the small intestines. It is probable that, if the seat of the obstruction were suspected to be about the middle portion of the colon, the opening may be most advantageously made on the right side near the commencement of this gut, which, as we are assured from the cases in which a preternatural anus has followed the sloughing of a portion of small intestine, would leave nothing to be apprehended on the score of an adequate nutrition. The effects of such an opening would be, that the contents of the gut would be evacuated from both sides of the wound ; and that portion, which was no longer distended by the passage of feces, would most probably contract, and perhaps follow, in time, a prevalent law in the animal economy, and become obliterated. In the case of my late patient, nothing like feculent matter appeared below the opening in the colon ; which seems sufficient to prove, in the first place, that the intestine, which is beyond the wound, will expel its contents at the wound, which was here some inches above the obstruction ; and, in the second, that the contents of the bowels, which are above the artificial opening, will be evacuated at this part, without passing beyond it, and forming a lodgment in that portion of intestine which is nearest the obstruction.

The eligibility of the operation is next to be considered on the second ground,—namely, how far the prolongation of life is desirable upon such terms ? On this point it may be remarked, that the great bulk of mankind consent to live under much greater evils than the one in question. The evils alluded to are generally of a moral kind : it would often be a charity to substitute for these one grand physical evil, as this operation will doubtlessly be considered, which should engross all the propensity that there is in human beings to torment themselves. Perhaps the advantages of an artificial anus may, nevertheless, not be accepted willingly upon so speculative a recommendation : for the further satisfaction, then, of those who may have occasion for such a facility, it may be observed, that the evil is not so great in reality as it is in apprehension. By means of stays, made to fit accurately above the hips, with a pad on one side, and buckles and straps in front, both the escape of feces, and the prolapsus of the intestine, may be effectually prevented. The bowels, by habit, will come to act pretty regularly once in twenty-four hours; and by habit, also, the attention to this part, which was at first disgusting, will become less repulsive, although, I fear, it will still amount to rather more than an interesting amusement, even if the patient should enjoy the advantage of having nothing else to do. I am of opinion, upon the whole, that an anus in the side is better than no anus at all: that, if life is desirable on other accounts, a convenience of this sort will not render it altogether intolerable.

*Bath; December* 3, 1821.

